# Occupational stress and associated factors among couriers: A Cross-Sectional study

**DOI:** 10.1186/s12889-025-24417-1

**Published:** 2025-09-24

**Authors:** Dexiang Zhu, Ruyun Bai, Meiqiong Guo, Liuzhuo Zhang, Xizhi Wang, Dafeng Lin, Shaofan Weng, Ming Zhang, Naixing Zhang

**Affiliations:** 1 Shenzhen Prevention and Treatment Center for Occupational Diseases, Shenzhen, 518020 Guangdong China; 2 Department of Occupational and Environmental Health, State Key Laboratory of Environmental Health (Incubating), School of Public Health, Tongji Medical College, Huazhong University of Science and Technology, Wuhan, 430030 Hubei China

**Keywords:** Couriers, Occupational stress, Work-related characteristics, Occupational health literacy

## Abstract

**Background:**

In all spheres of life, occupational stress has steadily emerged as a major influence on people's physiology and psychology. There is currently little research on couriers as a high-risk group for occupational stress.

**Objective:**

This study aims to explore the occupational stress levels among couriers in Shenzhen, China, and to analyze the contributing factors. The findings will provide a reference for managing and preventing occupational stress, thereby enhancing the occupational health and well-being of this workforce.

**Methods:**

This study enrolled 1500 couriers through convenience sampling from June 2022 to December 2023. Data on demographic, lifestyle, and work-related characteristics were collected through the online questionnaire survey. Occupational stress was assessed using the Job Content Questionnaire (JCQ). A logistic regression model was employed to calculate odds ratios (ORs) and 95% confidence intervals (CIs) to examine the relationships between occupational stress and work-related variables, such as tenure, job role, weekly hours worked, and occupational health literacy.

**Results:**

The prevalence of occupational stress among couriers was found to be 49.2%. Logistic regression analysis showed that couriers with a college degree or higher education level, alcohol consumption, irregular meal patterns, sedentary lifestyle, parcel delivery and collection couriers, working over 56 h per week, limited social support, and inadequate knowledge and practices regarding occupational health were more susceptible to occupational stress. Furthermore, individuals working more than 56 h weekly exhibited a 1.81 times higher risk of experiencing occupational stress compared to those working below 40 h per week [OR (95% CI) = 1.81 (1.17, 2.83)]. The couriers those deliver and collect parcels demonstrated a 1.62-fold higher risk of occupational stress compared with other couriers [OR (95% CI) = 1.62 (1.20, 2.19)]. Individuals with occupational health knowledge exhibited a 34% reduced risk of occupational stress [OR (95% CI) = 0.66 (0.53, 0.83)], while those practicing occupational health behaviors exhibited a 47% risk reduction [OR (95% CI) = 0.53 (0.39, 0.71)].

**Conclusions:**

Couriers are susceptible to elevated levels of occupational stress. Urgent action is required to address the problem of occupational stress among couriers. It is advised that the government, businesses, and trade unions work together to create a comprehensive "prevention-intervention-guarantee" system for occupational health protection.

## Introduction

Occupational stress is characterized by adverse physical and emotional reactions resulting from a mismatch between job demands and an individual's capabilities, resources, and needs [1]. In contemporary society, the prevalence of occupational stress has heightened. For instance, in a longitudinal study of public sector workers in 15 European nations, Rigó and colleagues discovered that the respondents' occupational stress rate increased from 28% in 1995 to 37% in 2015 [2]. It has been acknowledged as a major element influencing people's physical and mental health [3]. This type of stress is closely linked to the onset and progression of cardiovascular and cerebrovascular diseases [4, 5], metabolic diseases [6], musculoskeletal disorders [7] and depression [8]. Moreover, elevated workplace stress levels have been associated with increased accident rates, absenteeism, and staff turnover [9]. These outcomes can lead to decreased work productivity, elevated healthcare and social security costs, and substantial economic repercussions at both organizational and national levels.

The courier industry has experienced a significant surge in business volume due to the rapid expansion of the e-commerce sector. The workforce in the courier industry has grown substantially, with couriers emerging as a vital segment of the workforce in evolving employment structures. Data from the State Post Bureau [10] indicates that starting from 2024, China's annual express delivery per capita will exceed 100, with an average of over 5400 express deliveries per second. Furthermore, the peak daily business volume exceeds 729 million, and on November 17, 2024, the annual volume of express delivery business exceed 150 billion pieces for the first time. According to the Fifth National Economic Census [11], China hosts over 55 million logistics positions. Notably, the workforce in emerging sectors like instant delivery has expanded by over 50%, surpassing the average growth rate of urban employment during the corresponding timeframe.

According to Karasek's Demand-Control Model [12], occupational stress arises from the interaction between high job demands (e.g., time pressure, physical workload) and low decision control (e.g., limited autonomy). Prolonged exposure to this imbalance significantly elevates health risks. This framework effectively elucidates the stressful working conditions of couriers, who typically endure intensive delivery tasks (high demands) under algorithm-driven management systems that restrict autonomy (low control). Johnson's Social Support Theory [13] further posits that supportive resources in the workplace (e.g., supervisor support, team collaboration, institutional safeguards) can buffer the detrimental health effects of stress. In high-turnover occupations like courier services, insufficient social support may exacerbate job burnout and exhaustion. Collectively, these theories provide a structural lens for analyzing occupational health risks among couriers. Taken together, these frameworks suggest couriers may face elevated occupational stress risk due to workload intensity, constrained autonomy, and potential support deficits. Nonetheless, empirical evidence regarding the prevalence and influencing factors of occupational stress in this population remains limited [14, 15]. Systematic epidemiological studies are thus warranted to verify these relationships and inform targeted interventions.

Given the limited research on the occupational stress experienced by couriers, this study aims to investigate the current state of occupational stress among couriers and the contributing factors. The objective is to offer insights that can guide the prevention of occupational stress and enhance the overall occupational health and health of couriers.

## Methods

### Study populations

Employing convenience sampling, this cross-sectional study identified couriers through: (a) selection of two districts from Shenzhen's ten administrative regions; (b) comprehensive recruitment of all couriers within selected districts.

Sample size determination was based on the cross-sectional study formula:

$$ n = \frac{{Z^{2} P(1 - P)}}{d} $$

where *Z* = 1.96 (95% confidence level), *P* = 0.32 (anticipated occupational stress prevalence from pilot data [15]), and *d* = 0.025 (margin of error). This yielded a minimum requirement of 1388 participants; therefore, a target sample size of 1500 was deemed sufficient to meet the requirements of the study.

A total of 1500 couriers underwent physical examinations at the Shenzhen Prevention and Treatment Center for Occupational Diseases between June 2022 and December 2023. Inclusion criteria comprised individuals who were: (a) aged 18 years or above; (b) engaged in express operations for at least one year; (c) capable of effective communication and understanding to participate in the survey. Exclusion criteria encompassed participants who had recently encountered significant life stressors (e.g., bereavement, marital dissolution, traffic accidents) or reported a history of surgery, physical trauma, cancer, or mental illness. Ultimately, a total of 1442 participants were included for the final analysis. This study was approved by the Medical Ethics Committee of Shenzhen Prevention and Treatment Center for Occupational Diseases (LL2020-34).

### Questionnaire design and implementation

Prior to the formal study, we conducted a pilot test with 38 couriers in May 2022. Questionnaire refinements were implemented based on the findings. We developed standard semi-structured questionnaires incorporating online survey modules for occupational stress (JCQ), covariates (e.g. demographics, lifestyle and work-related characteristics) and occupational health literacy scales. During the formal data collection, which took place after the physical examinations, trained investigators guided the participants through the online survey. All responses were anonymised by linking them to medical records via unique participant codes that did not contain any personal identifiers.

### Assessment of occupational stress

In this study, occupational stress was evaluated using the Job Content Questionnaire (JCQ) [16], comprising 22 items across three domains: job demands (5 items), job control (9 items), and social support (8 items). Responses were recorded on a 4-point Likert scale, with positive responses (from 'strongly disagree' to 'strongly agree') scored from 1.0 to 4.0, and negative responses scored inversely. The assessment of occupational stress was determined by dividing the job demands dimension score by the job control dimension score, multiplied by 5 and divided by 9. A value above 1 on this scale indicates the presence of occupational stress. The social support dimension score was used to evaluate the level of social support of the subjects. Scores below 24.0 on this scale were classified low social support, while scores exceeding 24.0 indicated high social support. In this study, the Cronbach's α coefficients of the questionnaire, occupational stress and social support dimensions were 0.90, 0.84 and 0.90, respectively. This questionnaire has demonstrated good reliability and validity in previous research [6, 17], including large-scale studies conducted in Chinese populations, such as a study on coal miners (n = 1346) examining gene-environment interactions [17], and a study on petrochemical workers (n = 1683) analyzing associations with metabolic syndrome using dual occupational stress assessment models [6].

### Definition of covariates

The questionnaire included general demographic characteristics (e.g., age, gender, education level, marital status), lifestyle factors (e.g., smoking status, alcohol drinking status, physical activity), work-related characteristics (e.g., job category, length of employment, weekly working hours, occupational health literacy). Marital status was dichotomized as unmarried, married, and other (including divorced and widowed). Education levels were classified as junior school or below, vocational or high school, and college or higher education.

Smoking and alcohol drinking statuses were categorized as ever or never. Individuals were classified as smokers if they had smoked an average of ≥ 1 cigarette daily for more than six months in their lifetime; otherwise, they were considered as never smokers. Similarly, alcohol drinkers were treated as individuals who had drunk alcohol at least once a week for over six months. Otherwise, they were classified as never alcohol drinkers. Physical activity was designated for individuals engaging in regular physical activity for at least 20 min once a week or daily over the past year; otherwise, they were considered non-regular physical exercisers. Meal patterns were assessed by inquiring about the regularity of three meals. Meal patterns were classified into three categories based on the responses: regular, weekday regularity only, and irregular. Body Mass Index (BMI) was calculated as weight in kilograms divided by the square of height in meters (kg/m^2^). Following the Chinese obesity standard and the BMI distribution in this population [18], BMI was categorized as normal (18.5–23.9 kg/m^2^) and abnormal (< 18.5 kg/m^2^, ≥ 24 kg/m^2^).

Weekly working hours were interviewed and sorted into four categories [19]: ≤ 40 h, 40–48 h, 48–56 h, and > 56 h. Length of employment was divided into < 10 years or ≥ 10 years. Job roles among couriers were classified into three categories: parcel delivery and collection couriers, clerical management couriers and others (includes package sorters and shuttle drivers), based on job classifications and survey data.

The participants' occupational health literacy was assessed using the Occupational Health Literacy Questionnaire, adapted from the National Health Literacy Monitoring Questionnaire (2014 edition, NHLMQ) [20, 21] with input from experts and tailored to the characteristics of local occupational groups. The questionnaire includes four dimensions: occupational health knowledge (OHK, 4 items), occupational health attitude (OHA, 4 items), occupational health behaviours (OHB, 4 items) and occupational health skills (OHS, 4 items), totaling 16 items. Participants received 1 point for each correct answer and 0 points for incorrect responses. In this study, an overall occupational health literacy score of 80% or higher on the questionnaire was considered indicative of satisfactory overall occupational health literacy [22]. Similarly, if participants scored 3 or above on the OHK, OHA, OHB, and OHK questionnaires, they were deemed to possess adequate literacy in these areas. The questionnaire demonstrated a Cronbach's α coefficient of 0.70 in this study. Significance was found in the Bartlett sphericity test (*P* < 0.001), with a Kaiser–Meyer–Olkin value of 0.79.

### Statistical analysis

The Kolmogorov–Smirnov test was utilized to assess the normality of the data distribution. Results indicated that continuous variables in the study did not adhere to a normal distribution, and hence were presented as median values along with the 25th and 75th percentiles. Categorical variables were expressed as frequency (percentages). To compare the differences in basic information between individuals with and without occupational stress, a Chi-square test was performed. A logistic regression analysis was employed to identify the risk factors associated with occupational stress among couriers. Furthermore, logistic regression models were applied to estimate the associations of work-related characteristics with occupational stress by calculating odds ratios (ORs) and 95% confidence intervals (95% CIs). These associations were adjusted for age, gender, marital status, education level, BMI, smoking and drinking habits, physical activity, and dietary patterns. To evaluate the robustness of our findings, sensitivity analyse was conducted by repeating analyses on a randomly selected subset of 1338 participants, representing the minimum sample size required for sufficient statistical power. Statistical analyses were conducted using R version 4.4.1, with statistical significance set at a two-sided *P*-value of < 0.05.

## Results

### The characteristics of study population

In this study, a total of 1500 participants were initially recruited. After excluding 8 individuals with missing information, 1442 (96.13%) eligible participants were included in the final analysis. Among these participants, 1177 were male (81.6%) and 265 were female (18.4%). The predominant age group was 30–40 years old (51.0%), with a majority engaged in delivery and collection of parcels (61.4%). The majority of participants worked over 40 h per week (91.8%) (Table 1 General characteristics stratified by occupational stress status, Table 2 Work-related characteristics stratified by occupational stress status).

**Table 1:** General characteristics stratified by occupational stress status (n = 1442)

Variables	Total	Occupational stress		*P*^*a*^
	(n = 1442)	No	Yes	
		(n = 732)	(n = 710)	
Age (years), n (%)				0.703
< 30	395 (27.4)	207 (28.3)	188 (26.5)	
30–40	735 (51.0)	366 (50.0)	369 (52.0)	
≥ 40	312 (21.6)	159 (21.7)	153 (21.5)	
Gender, n (%)				0.078
Male	1177 (81.6)	584 (79.8)	593 (83.5)	
Female	265 (18.4)	148 (20.2)	117 (16.5)	
Marriage status, n (%)				0.071
Unmarried	489 (33.9)	268 (36.6)	221 (31.1)	
Married	916 (63.5)	444 (60.7)	472 (66.5)	
Other	37 (2.6)	20 (2.7)	17 (2.4)	
Education level, n (%)				0.012*
Junior school or below	137 (9.5)	86 (11.7)	51 (7.2)	
Vocational or high school	734 (50.9)	366 (50.0)	368 (51.8)	
College or above	571 (39.6)	280 (38.3)	291 (41.0)	
BMI, n (%)				0.076
Normal	681 (47.2)	363 (49.6)	318 (44.8)	
Abnormal	761 (52.8)	369 (50.4)	392 (55.2)	
Smoking status, n (%)				0.592
Never	852 (59.1)	427 (58.3)	425 (59.9)	
Ever	590 (40.9)	305 (41.7)	285 (40.1)	
Alcohol drinking status, n (%)				0.037*
Never	195 (13.5)	113 (15.4)	82 (11.5)	
Ever	1247 (86.5)	619 (84.6)	628 (88.5)	
Physical activity, n (%)				< 0.001*
No	993 (68.9)	447 (61.1)	546 (76.9)	
Yes	449 (31.1)	285 (38.9)	164 (23.1)	
Meal pattern, n (%)				< 0.001*
Regular	539 (37.4)	311 (42.5)	228 (32.1)	
Weekday regularity only	300 (20.8)	159 (21.7)	141 (19.9)	
Irregular	603 (41.8)	262 (35.8)	341 (48.0)	
Social support, n (%)				< 0.001*
Low	1175 (81.5)	553 (75.5)	622 (87.6)	
High	267 (18.5)	179 (24.5)	88 (12.4)	

Continuous variables were presented as mean ± SD or median (25th, 75th percentiles), and categorical variables were presented as n (%).BMI categories were defined as: Normal: 18.5–23.9 kg/m^2^; Abnormal: < 18.5 or ≥ 24.0 kg/m^2^

BMI, body mass index

^a^*P* values were calculated by using Chi-square test for categorical variable

^*^P < 0.05

**Table 2:** Work-related characteristics stratified by occupational stress status (n = 1442)

Variables	Total	Occupational stress		*P*^a^
	(n = 1442)	No	Yes	
Job category, n (%)				0.005*
Other	300 (20.8)	182 (24.9)	118 (16.6)	
Clerical management couriers	257 (17.8)	126 (17.2)	131 (18.5)	
Parcel delivery and collection couriers	885 (61.4)	424 (57.9)	461 (64.9)	
Length of employment (years), n (%)				0.003*
< 10	615 (42.6)	341 (46.6)	274 (38.6)	
≥ 10	827 (57.4)	391 (53.4)	436 (61.4)	
Weekly working hours (hours), n (%)				< 0.001*
≤ 40	118 (8.2)	76 (10.4)	42 (5.9)	
40–48	410 (28.4)	249 (34.0)	161 (22.7)	
48–56	297 (20.6)	140 (19.1)	157 (22.1)	
> 56	617 (42.8)	267 (36.5)	350 (49.3)	
Occupational health knowledge, n (%)				< 0.001*
No	579 (40.2)	257 (35.1)	322 (45.4)	
Yes	863 (59.8)	475 (64.9)	388 (54.6)	
Occupational health attitude, n (%)				0.520
No	157 (10.9)	84 (11.5)	73 (10.3)	
Yes	1285 (89.1)	648 (88.5)	637 (89.7)	
Occupational health behaviours, n (%)				< 0.001*
No	1202 (83.4)	574 (78.4)	628 (88.5)	
Yes	240 (16.6)	158 (21.6)	82 (11.5)	
Occupational health skills, n (%)				0.055
No	767 (53.2)	408 (55.7)	359 (50.6)	
Yes	675 (46.8)	324 (44.3)	351 (49.4)	
Overall occupational health literacy, n (%)				0.084
No	1029 (71.4)	507 (69.3)	522 (73.5)	
Yes	413 (28.6)	225 (30.7)	188 (26.5)	

Continuous variables were presented as mean ± SD or median (25th, 75th percentiles), and categorical variables were presented as n (%)

^a^*P* values were calculated by using Chi-square test for categorical variable

*P < 0.05

### Distribution characteristics of occupational stress

The prevalence of occupational stress among participants was 49.2% (710/1442). Table 1 (General characteristics stratified by occupational stress status) presents the general characteristics of the study participants stratified by occupational stress status, while Table 2 (Work-related characteristics stratified by occupational stress status) presents the work-related characteristics of the study participants stratified by occupational stress status. Significant disparities were observed in education levels, drinking status, physical exercise, meal pattern, job category, length of employment, weekly working hours, occupational health knowledge, occupational health behaviors and social support among participants with and without occupational stress (all *P* < 0.05). Compared to participants without occupational stress, those experiencing occupational stress exhibited higher education levels, alcohol consumption, irregular meal patterns, longer job tenure and weekly working hours, involvement in delivery and collection of parcels, lower levels of occupational health knowledge and practices, reduced engagement in regular physical exercise, and reported lower levels of social support (all *P* < 0.05).

### Multivariate logistic regression analysis of occupational stress

As shown in Table 3 (logistic regression model analysis for the association with the occupational stress), individuals with college degree or above [OR (95% CI) = 1.69 (1.11, 2.61)], alcohol drinking [OR (95% CI) = 1.48 (1.07, 2.06)], irregular meals [OR (95% CI) = 1.39 (1.07, 1.81)], employing in delivery and collection of parcels [OR (95% CI) = 1.46 (1.07, 1.98)], working more than 56 h per week [OR (95% CI) = 1.64 (1.05, 2.58)], and low social support [OR (95% CI) = 2.06 (1.53, 2.77)] displayed significantly heightened risks of occupational stress. Conversely, individuals who exercised regularly [OR (95% CI) = 0.57 (0.45, 0.73)], had sufficient occupational health knowledge [OR (95% CI) = 0.69 (0.55, 0.86)], and exhibited sufficient occupational health behaviors [OR (95% CI) = 0.57 (0.42, 0.78)] demonstrated significantly reduced risks of occupational stress.

**Table 3:** logistic regression model analysis for the association with the occupational stress

Variables	Coefficient estimate	Standard error	Z	*P*	OR (95% CI)
Education level					
Junior school or below					
Vocational or high school	0.27	0.21	1.31	0.189	1.31 (0.88, 1.98)
College or above	0.53	0.22	2.41	0.016*	1.69 (1.11, 2.61)
Alcohol drinking status					
Never					
Ever	0.39	0.17	2.36	0.018*	1.48 (1.07, 2.06)
Physical activity					
No					
Yes	− 0.56	0.13	− 4.43	< 0.001*	0.57 (0.45, 0.73)
Meal pattern					
Regular					
Weekday regularity only	0.04	0.15	0.26	0.797	1.04 (0.77, 1.41)
Irregular	0.33	0.14	2.44	0.015*	1.39 (1.07, 1.81)
Job category					
Other					
Clerical management couriers	0.33	0.19	1.79	0.073	1.40 (0.97, 2.01)
Parcel delivery and collection couriers	0.38	0.16	2.42	0.016*	1.46 (1.07, 1.98)
Weekly working hours (hours)					
≤ 40					
40–48	− 0.06	0.23	− 0.27	0.787	0.94 (0.60, 1.48)
48–56	0.41	0.24	1.72	0.085	1.51 (0.95, 2.41)
> 56	0.49	0.23	2.17	0.03*	1.64 (1.05, 2.58)
Occupational health knowledge					
No					
Yes	− 0.37	0.12	− 3.22	0.001*	0.69 (0.55, 0.86)
Occupational health behaviours					
No					
Yes	− 0.56	0.16	− 3.52	< 0.001*	0.57 (0.42, 0.78)
Social support					
High					
Low	0.72	0.15	4.79	< 0.001*	2.06 (1.53, 2.77)

^*^P < 0.05

### Associations of work-related characteristics with occupational stress

As shown in Table 4 (Association between work-related characteristics factors and occupational stress status), after adjusting for age, gender, BMI, marital status, and education level, employment tenure exceeding 10 years [model 1, OR (95% CI) = 1.37 (1.04, 1.79)] emerged as a risk factor for occupational stress among couriers. In comparison to working fewer than 40 h per week, working between 48 and 56 h weekly [model 1, OR (95% CI) = 1.95 (1.25, 3.08)] and exceeding 56 h [model 1, OR (95% CI) = 2.28 (1.49, 3.51)] posed as risk factors for courier occupational stress. Couriers engaged in delivery and collection of parcels demonstrated a 1.66-fold increased risk of occupational stress compared to those in other positions [model 1, OR (95% CI) = 1.66(1.24, 2.22)]. Moreover, clerical management couriers exhibited a 1.56-fold heightened risk of occupational stress relative to other job roles [model 1, OR (95% CI) = 1.56 (1.08, 2.24)]. Upon additional adjustment for smoking and drinking habits, meal patterns, and physical activity, the aforementioned associations persisted [model 2].

**Table 4:** Association between work-related characteristics factors and occupational stress status

Variables	Model 1		Model 2	
	OR (95% CI)	*P*	OR (95% CI)	*P*
Length of employment (years)				
< 10	Ref		Ref	
≥ 10	1.37 (1.04, 1.79)	0.024*	1.35 (1.03, 1.78)	0.029*
Weekly working hours (hours)				
≤ 40	Ref		Ref	
40–48	1.09 (0.71, 1.69)	0.701	0.97 (0.62, 1.51)	0.876
48–56	1.95 (1.25, 3.08)	0.004*	1.68 (1.07, 2.68)	0.027*
> 56	2.28 (1.49, 3.51)	< 0.001*	1.81 (1.17, 2.83)	0.008*
Job category				
Other workers	Ref		Ref	
Clerical management couriers	1.56 (1.08, 2.24)	0.017*	1.56 (1.08, 2.27)	0.018*
Parcel delivery and collection couriers	1.66 (1.24, 2.22)	< 0.001*	1.62 (1.20, 2.19)	0.002*
Occupational health knowledge				
No	Ref		Ref	
Yes	0.65 (0.53, 0.81)	< 0.001*	0.66 (0.53, 0.83)	< 0.001*
Occupational health attitude				
No	Ref		Ref	
Yes	1.09 (0.78, 1.52)	0.629	1.10 (0.78, 1.56)	0.578
Occupational health behaviours				
No	Ref		Ref	
Yes	0.48 (0.36, 0.64)	< 0.001*	0.53 (0.39, 0.71)	< 0.001*
Occupational health skills				
No	Ref		Ref	
Yes	1.17 (0.95, 1.45)	0.139	1.13 (0.91, 1.40)	0.264
Overall occupational health literacy				
No	Ref		Ref	
Yes	0.80 (0.64, 1.01)	0.065	0.88 (0.69, 1.12)	0.296

Model 1 was adjusted for age, gender, marriage status, education level and BMI. Model 2 further adjusted for smoking status, drinking status, physical exercise and meal pattern

^*^P < 0.05

As depicted in Table 4 (Association between work-related characteristics factors and occupational stress status), no significant correlations were observed between the overall score of occupational health literacy, occupational health attitude, occupational health skills, and occupational stress (all *P* > 0.05). After adjustment for common confounders, individuals with occupational health knowledge exhibited a 35% reduced risk of occupational stress [model 1, OR (95% CI) = 0.65 (0.53, 0.81)]. Similarly, those demonstrating occupational health behaviors displayed a 52% risk reduction [model 1, OR (95% CI) = 0.48 (0.36, 0.64)]. Subsequent to further control for smoking and drinking habits, meal patterns, and physical activity, the outcomes of the study remained largely unchanged [model 2]. The results presented in Table 5 (Association between work-related characteristics factors and occupational stress status (n = 1338)) demonstrate that the findings did not substantially change in the sensitivity analyses (Table 6).

**Table 5:** Association between work-related characteristics factors and occupational stress status (n = 1338)

Variables	Model 1		Model 2	
	OR (95% CI)	*P*	OR (95% CI)	*P*
Length of employment (years)				
< 10	Ref		Ref	
≥ 10	1.42 (1.07, 1.88)	0.016*	1.40 (1.06, 1.87)	0.019*
Weekly working hours (hours)				
≤ 40	Ref		Ref	
40–48	0.91 (0.58, 1.45)	0.694	0.89 (0.57, 1.42)	0.635
48–56	1.52 (0.94, 2.46)	0.088	1.50 (0.93, 2.43)	0.097
> 56	1.69 (1.08, 2.69)	0.024*	0.71 (1.08, 2.71)	0.022*
Job category				
Other workers	Ref		Ref	
Clerical workers	1.66 (1.13, 2.45)	0.011*	1.63 (1.10, 2.40)	0.015*
Pick-up and delivery workers	1.61 (1.18, 2.20)	0.003*	1.65 (1.21, 2.26)	0.002*
Occupational health knowledge				
No	Ref		Ref	
Yes	0.67 (0.53, 0.84)	< 0.001*	0.68 (0.54, 0.85)	< 0.001*
Occupational health attitude				
No	Ref		Ref	
Yes	1.05 (0.74, 1.50)	0.788	1.05 (0.74, 1.51)	0.774
Occupational health behaviours				
No	Ref		Ref	
Yes	0.52 (0.38, 0.71)	< 0.001*	0.52 (0.38, 0.70)	< 0.001*
Occupational health skills				
No	Ref		Ref	
Yes	1.15 (0.92, 1.43)	0.232	1.15 (0.92, 1.44)	0.218
Occupational health literacy				
No	Ref		Ref	
Yes	0.84 (0.66, 1.08)	0.171	0.85 (0.66, 1.09)	0.190

Model 1 was adjusted for age, gender, marriage status, education level and BMI. Model 2 further adjusted for smoking status, drinking status, physical exercise and meal pattern

^*^P < 0.05

**Table 6:** Association between work-related characteristics factors and occupational stress status (n = 1302)

Variables	Model 1		Model 2	
	OR (95% CI)	*P*	OR (95% CI)	*P*
Length of employment (years)				
< 10	Ref		Ref	
≥ 10	1.41 (1.06, 1.89)	0.020*	1.40 (1.04, 1.87)	0.025*
Weekly working hours (hours)				
≤ 40	Ref		Ref	
40–48	1.18 (0.74, 1.92)	0.485	1.06 (0.66, 1.74)	0.805
48–56	2.02 (1.24, 3.33)	0.005*	1.79 (1.09, 2.97)	0.024*
> 56	2.55 (1.61, 4.11)	< 0.001*	2.04 (1.27, 3.33)	0.004*
Job category				
Other workers	Ref		Ref	
Clerical workers	1.60 (1.16, 2.25)	0.008*	1.65 (1.13, 2.41)	0.009*
Pick-up and delivery workers	1.65 (1.26, 2.17)	0.004*	1.49 (1.09, 2.04)	0.013*
Occupational health knowledge				
No	Ref		Ref	
Yes	0.65 (0.51, 0.81)	< 0.001*	0.65 (0.51, 0.82)	< 0.001*
Occupational health attitude				
No	Ref		Ref	
Yes	1.08 (0.76, 1.55)	0.664	1.10 (0.76, 1.58)	0.622
Occupational health behaviours				
No	Ref		Ref	
Yes	0.46 (0.34, 0.63)	< 0.001*	0.51 (0.37, 0.69)	< 0.001*
Occupational health skills				
No	Ref		Ref	
Yes	1.19 (0.96, 1.49)	0.116	1.15 (0.92, 1.45)	0.215
Occupational health literacy				
No	Ref		Ref	
Yes	0.81 (0.63, 1.03)	0.087	0.88 (0.68, 1.13)	0.302

Model 1 was adjusted for age, gender, marriage status, education level and BMI. Model 2 further adjusted for smoking status, drinking status, physical exercise and meal pattern

^*^P < 0.05

## Discussion

Within the participant cohort, the prevalence of occupational stress was 49.2%, a notably higher figure than that reported among workers in conventional industries. Notably, a study from China involving 13,867 workers [23] revealed occupational stress prevalence rates of 3.3% in the supply enterprises, 10.3% in manufacturing enterprises, and 5.8% in transportation enterprises. Furthermore, a 2020 occupational stress survey across 12 industries in Japan [24] reported an occupational stress rate of 27.6%. Discrepancies in these findings could potentially stem from variations in research methodologies, participant selection criteria, and distinct regional occupational characteristics. However, the primary contributing factor appears to be the demanding working conditions encountered by couriers, characterized by heightened workloads, extended work hours, persistent time constraints in package deliveries, stressors related to traffic, and frequent interpersonal conflicts [14]. The amalgamation of these factors notably heightens their susceptibility to occupational stress in contrast to other worker groups.

In the present study, we observed a heightened likelihood of occupational stress among couriers with more than 10 years of service. This observation aligns with a study involving 1924 physicians and nurses in China, where a service tenure exceeding 10 years was linked to elevated stress levels [25]. Similarly, a population-based study in Spain reported significant associations between extended job tenure and an increased risk of occupational stress [26]. Individuals with shorter tenures are often characterized by higher levels of energy and enthusiasm compared to their longer-tenured counterparts, rendering them better equipped to cope with stress [27].

Furthermore, evidence indicates that couriers tasked with the delivery and collection of parcels are particularly vulnerable to occupational stress. This susceptibility can be elucidated by analyzing the time pressures inherent in managing the receipt and dispatch of parcels, coupled with the requisite customer interactions. Engaging in customer communication may lead to conflicts, contributing to strained interpersonal relationships that serve as stressors for occupational stress [28]. Additionally, among couriers, those in clerical management roles experience heightened levels of occupational stress compared to their non-managerial counterparts. A cross-sectional survey of 12,049 GPs across 11 countries demonstrated that GPs experiencing elevated levels of management burden were more likely to experience occupational stress [29]. For clerical management couriers, like data/order processors, stress is caused by a combination of high workloads, emotional depletion, and ongoing demands for accuracy. Since they operate under zero-tolerance accuracy conditions, where keeping uninterrupted focus is crucial to ensuring absolute precision in order information and fee settlements, they are always on edge to prevent any errors.

The study revealed that compared with working less than 40 h per week, working between 48 and 56 h per week, as well as exceeding 56 h per week, increased the risk of occupational stress among couriers. This finding aligns with the research conducted by scholars such as Gmayinaam [28] and others [30, 31]. The increased risk associated with extended work hours may stem from the reduction in employees' time available for rest and leisure activities. This limitation can impact sleep quality, with individuals experiencing poor sleep quality being more prone to occupational stress [32]. Furthermore, inadequate rest time can contribute to the adoption of unhealthy lifestyle behaviors such as alcohol consumption, smoking, and reduced physical activity, further heightening the risk of occupational stress [33, 34].

The result indicated that 28.6% of the participants had sufficient occupational health literacy. Among the four dimensions of occupational health literacy, the highest level of adequacy was observed in occupational health attitudes (89.1%), followed by occupational health knowledge (59.8%), with the lowest in occupational health behaviors (16.6%). These results are in line with occupational health literacy investigations conducted in Henan and Hebei provinces in China [21, 35], highlighting a prevalent understanding of knowledge but a deficiency in behavioral practices. This disparity could be attributed to a predominant focus on promoting and imparting occupational health knowledge among couriers in Shenzhen during training, potentially overlooking the enhancement of fundamental occupational health skills among workers. In the present study, a significant correlation was noted between adequate occupational health knowledge and behaviors and an increased prevalence of occupational stress. This finding aligns with the conclusions drawn by Michou [36], Fakhari [37] and Ying [38]. According to the occupational health literacy framework, couriers' practical coping mechanisms and cognitive control of stress are directly influenced by their knowledge and behavior. For instance, proactive health-related actions reduce physical and mental strain through focused interventions, while sufficient occupational health knowledge reduces anxiety by improving risk awareness. This demonstrates the strong relationship between work stress and the knowledge and behavior components. The two components of occupational health literacy—attitude and skills—as well as the overall score of occupational health literacy were not found to be related to occupational stress in this study. However, the attitude and skill components of occupational health literacy are constrained by external contextual factors. The core stresses that exist in the sector, such as strict performance requirements or structural workload limits, are not addressed by occupational health skills, despite their importance. As a result, they have no discernible effect on the level of stress at work. Furthermore, the four components of occupational health literacy—knowledge, attitude, competence, and behavior—combine to provide the total score. The knowledge and behavior dimensions' effective influence is often diminished by the attitude and skill dimensions' scores since they are unrelated to occupational stress.

It is important to acknowledge the limitations of this study. Primarily, Convenience sampling was used to choose two of Shenzhen's ten districts for this study, and a survey of all couriers in these two districts was administered. Convenience sampling is a non-probability sampling technique that could result in selection bias. Sensitivity analysis, however, showed that the pertinent factors affecting occupational stress did not alter, indicating that the study's findings are solid at the core parameter level. This has somewhat lessened the possibility that the research findings might be impacted by inadequate sample representativeness. It is unable to elucidate the causal relationship or temporal sequence of these factors in this cross-sectional study. The next stage will be to establish a cohort for subsequent studies. Additionally, while a wide range of work-related factors have been encompassed in this study, certain elements that may impact occupational stress, such as shift work, and rest intervals, were not addressed. The model of influencing factors for occupational stress remained unchanged after the research was redone and couriers who worked shifts were excluded from the study, mostly as package sorters and shuttle drivers in the study. Additionally, based on initial interviews, it was discovered that, aside from midday breaks, work breaks throughout working hours were largely the same for all couriers. Consequently, it can be said that shift work and breaks had little effect on the study's findings. The other elements of occupational stress will be examined in greater detail in future research. Finally, the occupational health literacy questionnaire used in this study has a skill dimension that is more focused on general occupational health issues and does not adequately capture skills that are specifically related to managing stress at work. The occupational health literacy questionnaire will be specifically optimized and revised going ahead, with modules addressing stress regulation and emotional management skills being added.

## Conclusions

In summary, urgent action is required to address the problem of occupational stress among couriers. It is advised that the government, businesses, and trade unions work together to create a comprehensive "prevention-intervention-guarantee" system for occupational health protection. First and foremost, the government should create occupational health regulations for couriers that outline the maximum number of hours they can work each week, incorporate occupational health metrics into the enterprise credit assessment system, and conduct mass media campaigns to promote occupational health across the country. Second, businesses should carry out their primary duties by enhancing training on occupational health literacy for couriers, establishing health stations at courier sites, and optimizing the order dispatch algorithm (which dynamically modifies delivery timeliness based on traffic and weather conditions). Thirdly, trade unions should take on a supervisory function in order to protect workers' rights and interests, create social and psychological support networks, and lessen psychological stress and occupational burnout.

## Data Availability

The datasets used during the current study available from the corresponding author on reasonable request.
